# *ROS1-ADGRG6*: a case report of a novel *ROS1* oncogenic fusion variant in lung adenocarcinoma and the response to crizotinib

**DOI:** 10.1186/s12885-019-5948-y

**Published:** 2019-08-05

**Authors:** Shuguang Xu, Wenxian Wang, Chunwei Xu, Xingliang Li, Junhui Ye, Youcai Zhu, Ting Ge

**Affiliations:** 1 Department of Respiratory Medicine, Ningbo Medical Center Lihuili Eastern Hospital, Ningbo, China 315010 People’s Republic of China; 2 Department of Chemotherapy, Zhejiang Cancer Hospital, Hangzhou, Zhejiang 310022 People’s Republic of China; 3 Department of Pathology, Fujian Cancer Hospital, Fujian Medical University Cancer Hospital, Fuzhou, Fujian 350014 People’s Republic of China; 4 Department of Thoracic Disease Diagnosis and Treatment Center, Zhejiang Rongjun Hospital, Jiaxing, Zhejiang 314000 People’s Republic of China; 5 Department of Respiratory, Sanmen People’s Hospital of Zhejiang, Zhejiang, 317100 People’s Republic of China; 6 Department of Respiratory, Ningbo Medical Center Lihuili Hospital, Ningbo, Zhejiang 315010 People’s Republic of China

**Keywords:** Lung adenocarcinoma, NGS, *ROS1* rearrangement

## Abstract

**Background:**

*ROS1* rearrangements are validated drivers in lung cancer, which have been identified in a small subset (1–2%) of patients with non-small cell lung cancer (NSCLC). To date, 18 fusion genes of *ROS1* have been identified in NSCLC. The ALK inhibitor (crizotinib) exhibits therapeutic effect against *ROS1*-rearranged NSCLC. Next-generation sequencing (NGS) technology represents a novel tool for *ROS1* detection that covers many fusion genes.

**Case presentation:**

A 55-year-old female with *EGFR* mutation (L858R) was diagnosed with lung adenocarcinoma, who was responsive to first-generation EGFR-tyrosine kinase inhibitor (TKI). Afterwards, she developed acquired resistance accompanied with a *ROS1* rearrangement. A NGS assay showed that the tumor had a novel *ROS1-ADGRG6* rearrangement generated by the fusion of exons of 1–33 of *ROS1* on chr6: q22.1 to exons of 2–26 of *ADGRG6* on chr6: q24.2. The patient was obviously responsive to crizotinib.

**Conclusion:**

We firstly identified *ROS1-ADGRG6* fusion variant in NSCLC by NGS, which should be considered in further ROS1 detecting assays.

## Background

Morbidity and mortality of lung cancers has been gradually increased during the past several decades [1]. The ROS proto-oncogene 1, receptor tyrosine kinase (*ROS1*) gene is proved to be a valuable therapeutic target in patients with non-small cell lung cancer (NSCLC). It has been established that solid tumors have unstable genomes, and many fusions are caused by genetic instability. The prevalence of *ROS1* rearrangements is estimated in 1–2% of NSCLC patients [2]. Up to date, a total of 18 *ROS1* fusion genes have been reported in lung cancer, including *CD74*, *SLC34A2* and *GOPC* [3–5]. All *ROS1* gene fusions harbor the ROS1 kinase domain, with *CD74-ROS1* being the most common fusion partner. Studies have shown that these alterations frequently lead to activation of signaling pathways that are critical for carcinogenesis and progression, such as MAPK and PI3K/AKT pathways. Moreover, these fusions play a prognostic role in lung cancer [6]. For example, *ROS1* fusion-positive patients with lung cancer have poorer disease-free survival (DFS) than those fusion-negative patients [7].

Crizotinib is an anaplastic lymphoma kinase (ALK)/ROS1/MET inhibitor. Based on efficacy and safety data from a clinical trial, crizotinib has become the first targeted agent approved by the FDA for the treatment of advanced *ROS1*-rearranged NSCLC [8, 9]. In addition to FISH, IHC, and PCR, next-generation sequencing (NGS) has emerged as a new diagnostic approach for detection of *ROS1* rearrangements in recent years.

In this case, we identified a novel *ROS1* fusion gene in a lung adenocarcinoma patient. We also report that the patient was sensitive to treatment with ROS1-directed tyrosine kinase inhibitors (TKIs).

## Case presentation

**Fig. 1:**
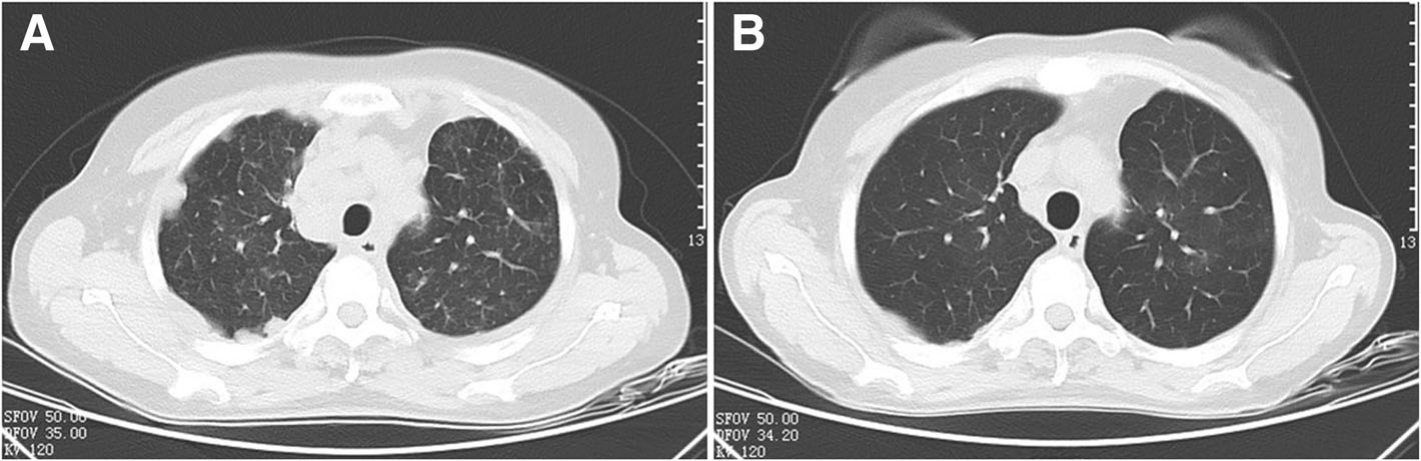
Computed tomography (CT) scans before (**a**) and after (**b**) crizotinib therapy

**Fig. 2:**
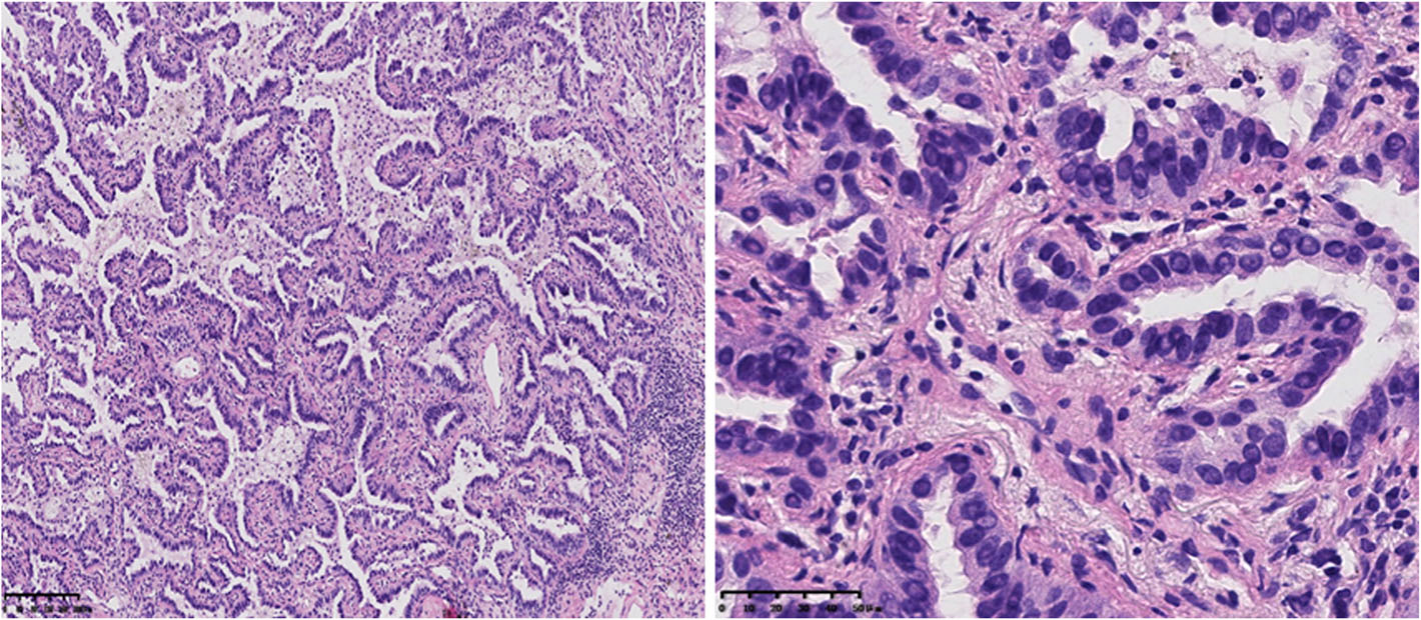
Surgery of brain tumor showed adenocarcinoma lung cancer (HE × 10, left; HE × 40, right)

**Fig. 3:**
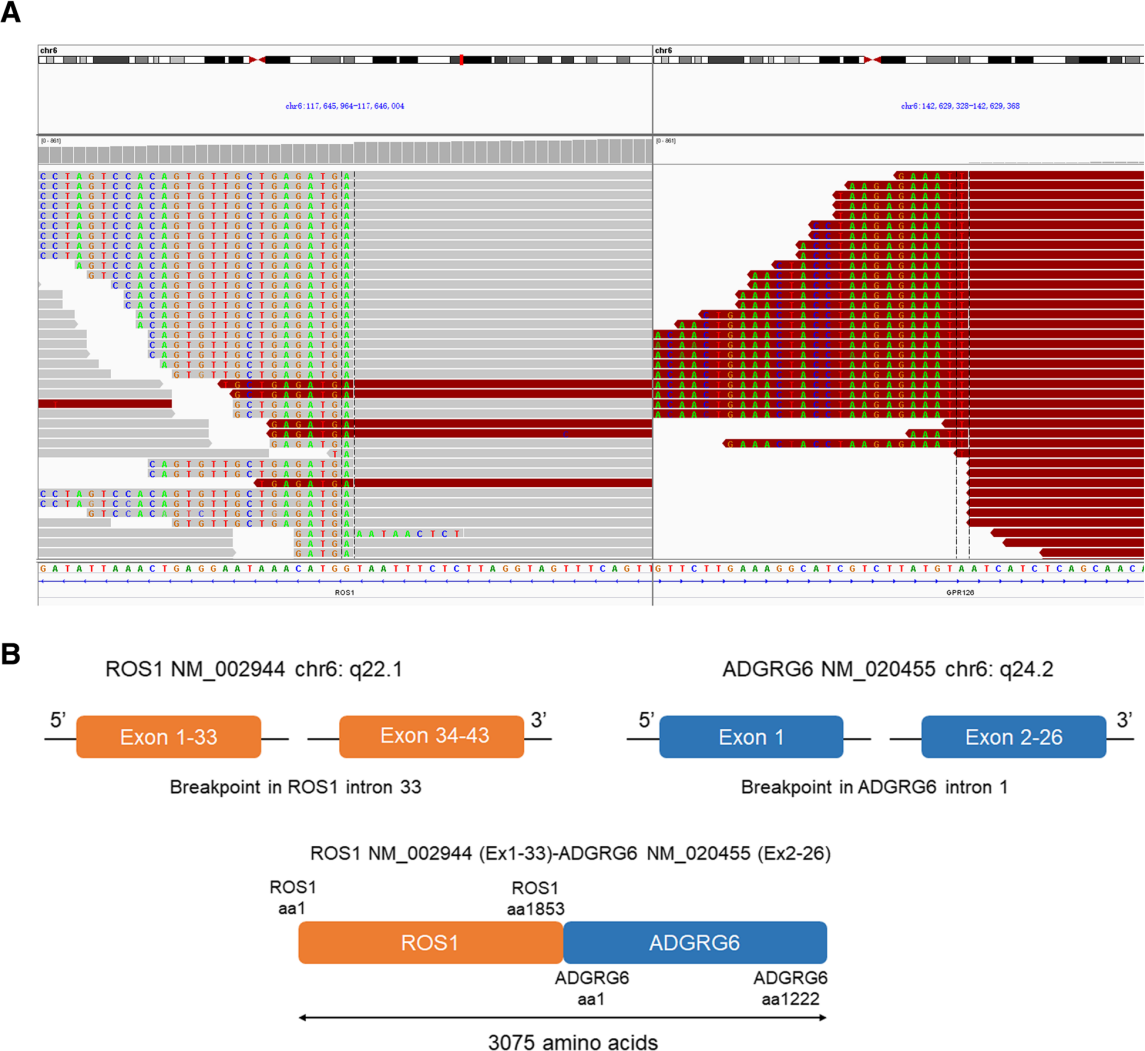
*ROS1-ADGRG6* fusion is clinically present. **a**, Integrative Genomics Viewer snapshot of *ROS1-ADGRG6*. Breakpoints were localized at 6q22.1 and 6q24.2, respectively. Soft-clipped bases match one another in reverse complementarity. **b**, Schematic representation of the *ROS1-ADGRG6* fusion protein domain structure. Orange, *ROS1*; blue, *ADGRG6*. The fusion protein is 3075 amino acids in length

A 55-year-old female was referred to our hospital in April 2016 with a 2-month history of cough and phlegm. A computed tomography (CT) scan revealed multiple nodules in the left lower lung (Fig. 1a). She underwent thoracoscopic surgery for radical resection of lung tumors. Hematoxylin and eosin (H&E) staining revealed a typical morphology for adenocarcinoma cells (Fig. 2). The patient relapsed in November 2016 and was initially treated with gefitinib due to detection of an *EGFR* mutation (L858R) without ROS1 fusion by the captured targeted next-generation sequencing 381 panel. Although a decrease in tumor size was obtained in a short-time period, long-term effects were not achieved. Subsequently, she underwent chemotherapy (pemetrexed and carboplatin for 6 cycles, pemetrexed alone for 2 cycles) in December 2016. Then, the patient was treated with oral afatinib administration in August 2017, and combined treatment with docetaxel and carboplatin for 5 cycles in November 2017. However, the response was inadequate. After three months, chest CT scan images indicated an increase in tumor size. A NGS analysis of the hydrothorax revealed a novel *ROS1-ADGRG6* rearrangement, as shown in Fig. 3a (3D Medicines, Shanghai China). This novel *ROS1-ADGRG6* rearrangement was generated the fusion of exons of 1–33 of *ROS1* on chr6: q22.1 to exons of 2–26 of *ADGRG6* on chr6: q24.2. The predicted ROS1-ADGRG6 protein product contained 3075 amino acids comprising the N-terminal amino acids 1–1853 of *ROS1* and C-terminal amino acid 1–1222 of *ADGRG6* (Fig. 3b). Thus, the patient received oral crizotinib therapyin April 2018. After 1 month, a chest CT scan showed a decrease in tumor size and the patient achieved a partial response to crizotinib (Fig. 1b). During crizotinib therapy, there were no adverse events, such as rashes, cordis damage, and gastrointestinal reactions. Thus far, the disease remains stable and she is still under treatment with crizotinib after 6 months.

## Discussion and conclusion

Currently, 18 fusion partners of *ROS1* fusions have been reported in lung cancer. A functional investigation has shown the oncogenic potential of *ROS1* fusions. For example, *ROS1* fusions results in transformation of NIH3T3 in vitro and tumorigenicity in vivo [10, 11]. Transgenic mice harboring *EZR-ROS1* in the lung alveolar epithelial cells develop bilateral lung adenocarcinomas [12, 13]. Indeed, *ROS1-ADGRG6* rearrangement has not been previously reported in lung cancer, thus this is the first report of a novel *ROS1* fusion variant. Given that the patient was initially responded to gefitinib, but later developed acquired resistance, we proposed this novel *ROS1* fusion may be responsible for the acquired EGFR-TKI resistance.

Adhesion G protein-coupled receptor G6 (*ADGRG6* [also referred to as GPR126]) is located on chromosome 6q24.2 and contains 28 exons, while ROS1 is located on chromosomes 6q22.1. ADGRG6 is a member of the adhesion G protein-coupled receptor family, which consists of a seven-transmembrane domain and a long N-terminal region involved in cell adhesion [14, 15]. Thus, it remains to be determined whether or not patients with *ROS1*-rearranged lung cancer and the *ROS1-ADGRG6* fusion exhibit unique clinicopathologic manifestations, such as metastasis.

Although crizotinib was approved to treat advanced lung cancer with ROS1 rearrangement, there are currently no approved companion diagnostic assays to detect *ROS1* rearrangements in NSCLC. Traditional methods (including FISH and IHC) have limitations, such as they both depend on diagnostic expertise. Another diagnostic method, i.e., RT-PCR, is unable to detect novel chromosomal rearrangements [15, 16]. By contrast, NGS allows for detection of both known and previously unreported *ROS1* rearrangements, as in this case.

Malignant pleural effusions (MPEs) are often present in advanced lung cancer patients. Given that MPEs contain tumor cells and biomarkers, they are considered to be an alternative to tumor tissues for detection of genetic mutation and fusions. FISH and RT-PCR have been successfully applied to detect *EGFR* mutations and *ALK* rearrangements in MPEs [7, 17]. In our case, the *ROS1* fusion was detected in a MPE using NGS, suggesting that evaluation of a MPE represents an alternative and feasible method to detect gene fusions in NSCLC.

There are some limitations in our present study. Firstly, this is only a case report and more cases are needed to analyze the correlation of *ROS1-ADGRG6* and clinical parameters, such as overall survival and progression-free survival. Secondly, the biological function of *ROS1-ADGRG6* should be further investigated using cell lines and animal models after molecular manipulation of ROS1-ADGRG6.

In summary, the present case indicated that *ROS1-ADGRG6* fusion may underlie the acquisition of resistance against EGFR-TKI and suggested an important role for the diagnostic application of NGS in precision medicine.

## Data Availability

For patients’ privacy, the patient information is publicly inaccessible.

## Abbreviations

ADGRG6: adhesion G protein-coupled receptor G6
ALK: Anaplastic lymphoma kinase
NGS: Next-generation sequencing
TKIs: Tyrosine kinase inhibitors
